# Construction and Initial Psychometric Validation of the Morana Scale: A Multidimensional Projective Tool Developed Using AI-Generated Illustrations

**DOI:** 10.3390/jcm14197069

**Published:** 2025-10-07

**Authors:** Tytus Koweszko, Natalia Kukulska, Jacek Gierus, Andrzej Silczuk

**Affiliations:** 1 Department of Community Psychiatry, Faculty of Health Sciences, Medical University of Warsaw, 02-091 Warsaw, Poland; andrzej.silczuk@wum.edu.pl; 2 Prof. Jan Mazurkiewicz Psychiatric Student Research Group of the Medical University of Warsaw, Faculty of Health Sciences, Medical University of Warsaw, 02-091 Warsaw, Polandjgierus@gmail.com (J.G.)

**Keywords:** suicide, psychometrics, projective techniques, mental health, artificial intelligence

## Abstract

**Background/Objectives**: Psychoanalytic theories of destructiveness highlight its deep, unconscious origins tied to primal emotional and motivational mechanisms. Traditional psychiatric models of suicidal risk assessment focus on classic risk factors, limiting diagnostic and intervention approaches. This study examines the neuropsychoanalytic foundations of destructive tendencies, integrating sublimation and evolutionary motivational systems, redefining their role in the destruction process. **Methods**: A total of 480 AI-generated illustrations were assessed for interpretative accuracy. The final set was used in an online projection task with 204 respondents. Analyses included factorial exploration of the structure of the tool, assessment of psychometric properties (Cronbach α, ROC, AUC), logistic regression and analysis of intergroup differences. **Results**: Factor analysis identified eight subscales. Six of the eight factors showed thematic resemblance to Panksepp’s emotional systems, although this interpretation remains theory-driven and requires empirical validation. The remaining two—pursuit of destruction and its sublimation—extend beyond natural evolutionary mechanisms. Destructiveness was best explained by depression and psychological pain (OR = 1.39, 95% CI [1.26–1.52]), aggression and impulsivity (OR = 1.68, 95% CI [1.36–2.06]), and anxiety and a sense of threat (OR = 1.55, 95% CI [1.27–1.90]). Key predictors of destruction sublimation were curiosity (OR = 3.15, 95% CI [2.43–4.09]), closeness and love (OR = 3.43, 95% CI [2.48–4.76]), and pleasure and fun (OR = 3.08, 95% CI [2.26–4.20]). Analyses showed higher levels of destructiveness in individuals receiving psychological or psychiatric support, those with prior diagnoses, and students compared to employed individuals. **Conclusions**: Results indicate high reliability (Cronbach’s α > 0.87) and discrimination among internal subscale-defined groups (ROC > 0.7), supporting the tool’s potential in assessing destructive and sublimation tendencies within a neuropsychoanalytic framework. Future studies will explore its external validity and clinical applications.

## 1. Introduction

Suicide is a multidimensional phenomenon involving various groups of specialists. Clinical understanding of suicidal behavior is based on the analysis of the interaction of psychological, biological, social and environmental factors. According to the World Health Organization, more than 720,000 people take their own lives every year, making suicide the third cause of death in the 15–29 age group [1]. In Poland, about 15 people take their own lives every day, and the number of suicide deaths is twice as high as deaths caused by road accidents [2].

From a health care perspective, effective diagnosis of individuals who reveal suicidal risk is critical in the context of efficient identification of at-risk individuals who require preventive or curative interventions [3,4,5].

Psychological questionnaire methods support the diagnosis of suicidal risk. Popular diagnostic tools commonly used in clinical practice include the Beck Scale for Suicide Ideation (BSS), Suicide Behavior Questionnaire (SBQ-R), Columbia Suicide Severity Rating Scale C-SSRS or Suicidality Scale (SS) [6]. New method proposals are also being developed, such as the ‘open source’ Suicidality Scale [7].

The available traditional scales designed to assess suicide risk, despite their high psychometric validity, have documented limitations. These include reduced predictive validity, vulnerability to intentional distortion, and overreliance on self-report formats, which may obscure clinical judgment and contextual understanding [8].

In order to gain access to more subtle mental processes and deeper unconscious content, the projective method was developed and used in the course of the presented study, enabling the exploration of the unconscious. Within the framework of psychoanalytic theory, the unconscious is a mental area into which unaccepted content is dissociated. Thus, traditional research, which gives access to informed content, is inevitably associated with limitations affecting the quality and reliability of the assessment. The projective form, by assessing the automatic response to a stimulus, makes it possible to project hidden impulses, conflicts, emotions and drives, with minimized resistance from the subject [9,10,11]. This form of examination opens up a unique perspective on the diagnosis of suicide risk.

### 1.1. Unconscious Processes as the Foundation of Projective Diagnostics

Unconscious processes influence cognition, emotion, and behavior despite remaining outside awareness. According to Sigmund Freud’s concept, thanks to the mechanism of repression, unaccepted fears, desires and psychological conflicts are repressed to the unconscious, they shape personality, emotions, and behavior. On the other hand, thanks to the mechanism of projection, what is hidden in the area of the unconscious can be projected onto external objects. In this way, the person who first denies and then projects does not expose themselves to the discomfort resulting from identification with unwanted content. It is on this mechanism that psychological projective methods are based [12,13].

### 1.2. Freud’s Death Drive as a Theoretical Basis for Self-Destructiveness

According to the Freudian dynamic model it, two dominant drives are at the root of thoughts, feelings and behaviors: life and death. The drive for life (libido) serves the survival of the individual and the species. On the other hand, the death drive (Thanatos, or destrudo) is focused on destruction and decay, a return to a state of non-existence. Its occurrence can have both positive and negative dimensions. It may manifest destructively or be redirected through sublimation. The process of transforming aggressive and destructive impulses into socially acceptable and even desirable actions occurs within the mechanism of sublimation [14,15].

Therefore, the presence of the death drive is an integral element of the human psychic world. However, its severity and impact on the ability to function properly and steadily vary depending on a number of factors [16]. Based on classical psychodynamic theory, Jaccard and Jacoby developed a model to explain the role of the death drive in suicidal behavior. As a result of the loss of the emotional object of love, the ego experiences weakening. Hostile impulses are redirected to the ego itself, and hateful feelings towards the lost object begin to be experienced as their own defect. In the face of additional adverse circumstances, this can push to fantasies of death and even suicide [17,18].

Projective methods have the potential to stimulate unconscious processes through the use of ambivalent and symbolic stimuli. In this way, thoughts, emotions, desires and conflicts between the drive for life and death can be revealed, which in other circumstances would not be expressed. Emotionally charged images may evoke unconscious responses in individuals experiencing psychological tension or conflict [11].

### 1.3. Neurobiological Basis of Unconscious Processes

According to Paul D. MacLean’s theory, the human brain can be divided into three systems, developed at different stages of evolution. These structures include the reptilian brain, the limbic brain, and the neocortex. From the perspective of emotional reactions, the oldest primitive reptilian brain is responsible, m.in other things, for feelings of fear and sexual desire. The limbic system is the center of emotions, social relationships, and memory, but also of unconscious processes that are associated with risky behaviors. The youngest structure, the neomammalian brain, is responsible for thinking, consciousness, language and planning [19]. These evolutionarily developed structures determine the functioning of man. The environmental situations and circumstances experienced by the individual, as well as the images presented in projective diagnostic methods, cause the activation of mental processes related to the limbic brain. It is the focus on this area that is the subject of our research interest.

### 1.4. Suicide Risk Factors

In suicide prevention and clinical practice, an important role is played by the efficient diagnosis and identification of clinical risk factors. Diagnostic assessment of the full picture of the phenomenon requires a multidimensional approach, taking into account factors such as depression, aggressiveness, impulsivity, anxiety, psychological pain and suicidal thoughts.

#### 1.4.1. Depression

Depression is a mental disorder that particularly predisposes to suicidal behavior [20,21]. Scientific evidence points to a complex etiopathogenesis of suicide in depression. Risk factors include genetic aspects, exogenous and endogenous stressors (interpersonal, occupational, financial), psychiatric disorders, epigenetic alterations, dysfunctions of the hypothalamic–pituitary–adrenal axis, abnormalities in the neurotransmitter system (especially serotonin), lipid profile, neuroimmune biomarkers, and neurotrophic factors and other neuromodulators [22]. The theoretical basis of depression as a driving force for suicide is a cognitive-behavioral model, such as Beck’s theory of hopelessness [23,24].

#### 1.4.2. Aggression

Suicide is an act of aggression, a murder committed on oneself. Aggression, along with impulsivity, stressful life events, mental disorders and a sense of hopelessness, are clinical suicidal risk factors. This is described in various theories explaining the process of suicide, such as the Interpersonal Theory of Suicide [25,26], the Cry of Pain Model, and the Stress-Diathesis Model [27].

#### 1.4.3. Impulsivity

Studies confirm the relationship between impulsivity and the risk of engaging in self-destructive behaviors, including suicidal behavior. From the neurobiological level, emotional dysregulation plays an important role here, which can lead to risky activities, including those of a self-aggressive nature. Impulsivity plays an essential role in sudden, violent, and unplanned suicide attempts [28].

#### 1.4.4. Anxiety

Anxiety disorders are both an independent risk factor for later suicidal thoughts and behaviors, as well as a predictor of suicide attempts in people with mood disorders [29]. Chronic anxiety can significantly increase psychological tension and foster the desire to take one’s own life [30]. Studies of patients admitted to a general psychiatric ward during the year showed that patients diagnosed with F40–F49 had the highest rate of suicide attempts before admission, compared to patients with other diagnoses [31].

#### 1.4.5. Psychache (Psychological Pain)

According to Edwin Shneidman’s concept of psychological pain, suicide is the result of unbearable mental suffering resulting from the unmet psychological needs [32]. Shneidman defined psychological pain as “the experience of anguish, suffering, and negative emotions such as terror, despair, fear, grief, shame, guilt, lack of love, loneliness, and loss” [33]. Suicide, therefore, is not so much a desire to take one’s own life, but rather a way to break the stream of unbearable pain [34]. Bolger described psychological pain in the context of a traumatic event that destroys an individual’s personal identity and affects relationships with other people. The result is emotional distress, causing shattering, wounding, diffusion of identity, disconnectedness, and intensification of negative mental states. Various definitions of psychological suffering boil down to understanding it as a persistent, unbalanced, and unpleasant feeling resulting from a negative evaluation of oneself or the loss experienced [35].

#### 1.4.6. Fantasies About Death

Suicidal thoughts can take many forms and intensities, from fleeting thoughts of death to specific plans to take one’s own life [36]. From a clinical perspective, they are a warning signal. Research confirms that suicidal thoughts are a predictor of later suicidal actions. The relationship between suicidal thoughts and suicide is described by theoretical concepts such as Shneidman’s theory of psychological pain [32], Beck’s theory of hopelessness [23], and Joiner’s interpersonal theory [25]. Integrating the above perspectives, it can be assumed that suicidal thoughts are born out of intense, unbearable suffering, accompanied by a pessimistic perception of reality and disturbed relationships with other people.

### 1.5. Protective Factors of Suicide

Protective factors serve as a crucial safeguard against suicidal thoughts and behaviors. While they play an important role in mitigating suicide risk, they may not be sufficient to fully counteract severe acute risk factors. Assessing these protective elements is essential in suicide prevention. Posner highlights the significance of social support, access to psychological care, and effective coping mechanisms for managing stress [37].

Protective factors can be categorized into internal and external influences, both of which contribute to resilience and suicide prevention. Internal protective factors include an individual’s ability to cope with stress, frustration tolerance, religious or spiritual beliefs that discourage self-harm, and fear of death or the act of suicide itself. Additionally, identifying clear reasons for living serves as a strong internal safeguard against suicidal thoughts [38].

On the other hand, external protective factors encompass broader social, familial, and environmental influences that contribute to suicide prevention. These factors include cultural, spiritual, and moral attitudes opposing suicide, a sense of responsibility for children or loved ones, emotional attachment to beloved pets, a supportive social network of family and friends, and positive therapeutic relationships. Engagement in work or education further strengthens psychological resilience and personal stability [39,40,41]. Moreover, research shows that protective factors moderate the effects of stress on depression and suicidal behavior [42].

### 1.6. The Role of Artificial Intelligence in the Diagnostic Process

Given the complexity and interplay of suicide risk and protective factors—many of which are difficult to capture through traditional assessment—recent research has turned to technological solutions such as machine learning (ML) and artificial intelligence (AI) to enhance diagnostic precision and predictive capacity [43]. This is consistent with the trend of recent years, where the growing role of artificial intelligence algorithms in medical data analysis is observed [44].

The Stanford Institute for Human-Centered Artificial Intelligence defines artificial intelligence as the ability to learn and apply appropriate techniques to solve problems and achieve goals in a changing and uncertain world. A key pillar of AI is machine learning, which allows computers to improve their operations based on experience or database analysis [45]. AI enables the analysis of large data sets with exceptional precision, identifying even subtle factors that may indicate an increased risk of suicide. An example is the use of machine learning methods to analyze the words, actions, and gestures of adolescents, which allowed them to effectively distinguish between people with suicidal thoughts and those who did not [46]. Machine learning models based on retrospective data analysis, for example in the case of patients with multiple sclerosis, have shown that creating predictive models for specific groups of patients can improve the accuracy of predictions, providing information even several years before a potential suicide attempt [47]. In a study of soldiers using outpatient therapy, the use of machine learning software allowed for the identification of a combination of variables that, when combined, showed greater sensitivity in determining individuals at high risk of suicide than many previous models [48].

Machine learning also enables better personalization of treatments and interventions. For example, in the treatment of Treatment-Resistant Depression (TRD), the use of a machine learning algorithm has generated an efficient model with an accuracy of up to 75% in predicting treatment outcomes, thus exceeding the current predictive capabilities of clinical assessment [49]. In addition, it has been observed that the use of short, automated interventions in digital applications to reduce barriers to access to psychological help can increase the use of this type of support by people in mental crisis—even in the case of people experiencing severe mental stress [50].

The ongoing boom in the emergence of new solutions for the detection of suicide risk includes, for example, activities focused on people using social media based on text data, but also modern solutions based on the analysis of text and vocal pattern [51]. In a meta-analysis by Ehtemam et al. [52], the authors confirmed the effectiveness of machine learning algorithms in uncovering hidden connections and providing precise predictions about suicide risk, depending on the appropriate selection of algorithms.

### 1.7. Aim of the Study

The aim of the presented study is to develop and initially validate a multidimensional scale for suicide risk assessment, which, thanks to its projective nature and simplicity, can be an effective screening tool for early identification of people at risk of suicide who require further intervention and support.

## 2. Materials and Methods

### 2.1. Study Design and Setting

This study uses artificial intelligence to generate illustrations using advanced machine learning methods that process input data and create unique images. In this way, symbolic and ambiguous projective stimuli were created, which were aimed at activating unconscious processes in the subjects related to repressed emotional conflicts and assumed risk factors: depression, aggressiveness, impulsivity, anxiety, psychological pain and suicidal thoughts. The use of artificial intelligence to create illustrations allowed for the standardization and replicability of stimuli, as well as the identification of symbols with which a person in emotional crisis can identify. Using the capabilities of artificial intelligence, the authors of the presented method decided to use illustrations created by AI, which are the key to universal patterns of thinking and the collective unconscious, referring to archetypal images and common human experiences. This makes it possible to reach deep-rooted emotions and thoughts that are not always available at the level of conscious processing. The graphics reflect the common elements of the human psyche, which allows for a more universal and effective approach to suicide risk assessment. Thus, the method aims to enable a more precise and holistic recognition of the risks associated with suicide. In the further part of the publication, the process of construction and initial validation of a multivariate projective scale for the assessment of suicide risk is presented.

The survey was conducted electronically using an algorithm that sent an invitation to participate in the study via social media to people who were interested in the subject of mental health. The recruitment period for this study commenced on 5 January 2025, and concluded on 10 February 2025. Participation in the study was voluntary and anonymous, and the subjects did not receive any benefits for participation and could discontinue their participation at any time. At the beginning, the respondents were informed about the form and purpose of the study, and sending the completed questionnaire was equivalent to giving informed consent to participate in the study and the subsequent use by the researchers of the obtained data to prepare a scientific publication describing its course and results. In the first part of the survey, the participants were asked to answer about basic general (sociodemographic) data, use of psychotherapy, previous psychiatric treatment and diagnosis, as well as its impact on current well-being.

In the second part of the survey, respondents were asked to choose any number of displayed illustrations that best described their well-being over the past two weeks. This reference period was chosen to align with established clinical standards in mental health assessment, such as the Patient Health Questionnaire (PHQ-9) [53] and Beck Depression Inventory-II (BDI-II) [54], ensuring reliable recall while capturing recent fluctuations in emotional state and suicide risk.

The scale was given the name Morana Scale. The name comes from the name of the Slavic goddess who was responsible for the death of vegetation in winter and the rebirth of nature in spring. Moran’s name derives from the Proto-Indian root mar- or mor-, which means death [55,56].

### 2.2. Competent Judges Procedure

At the initial stage of the construction of the Morana Scale, the researchers created 480 illustrations assigned to 12 theoretical reference frameworks that referred to risk factors and protective factors of suicide. Six clinical risk scales and the same number of opposing protective scales included the following pairs of theoretical factors: fantasies about death—desire for life, impulsivity—reflexivity/balance, depression—vitality/affirmation of life, mental pain—pleasure/relief/bliss, anger/aggression—composure/calmness, and anxiety—sense of security.

A total of 480 images were generated using a virtual image creator powered by an artificial intelligence illustration model (licensed version of Fotor Pro Plus). For each of the 12 categories, researchers manually entered English-language prompts directly into the generator interface. These prompts were identical to the category labels and included: fantasies about death; desire for life; impulsivity; reflexivity/balance; depression; vitality/affirmation of life; mental pain; pleasure/relief/bliss; anger/aggression; composure/calmness; anxiety; sense of security. Each prompt yielded 40 illustrations. No seed values were used, and no negative prompts as “no text” or “no face”) were applied. All prompts were affirmative and category-specific, designed to evoke the intended emotional or conceptual domain. In order to limit the number of illustrations and select those that most accurately relate to each category, the images were presented to 15 experts, who took on the role of competent judges. The expert panel consisted of 15 professionals with at least 10 years of clinical experience in psychiatry, clinical psychology, or psychotherapy. Six held doctoral degrees (PhD), nine were licensed psychiatrists, and six were clinical psychologists. All participants had completed formal specialization in their respective fields and were actively engaged in suicide risk assessment. The subjects rated each of the illustrations on a 5-point Likert scale in relation to the category they were supposed to represent. To assess the consistency of ratings provided by competent judges, we computed the Intraclass Correlation Coefficient (ICC) using a two-way random effects model with average measures (ICC(2,k)). Ratings were collected from 15 independent judges across 480 AI-generated illustrations, grouped into 12 predefined thematic categories (40 images per category). ICC was calculated separately for each category using the pingouin statistical package in Python. All rating data were complete, with no missing values. All ICC values were statistically significant (*p* < 0.001), ranging from low agreement in categories such as impulsivity (ICC = 0.14, 95% CI [0.07, 0.24]) to moderate levels observed for anger/aggression (ICC = 0.43, 95% CI [0.30, 0.59]). These results reflect the varying degrees of conceptual clarity and visual salience across categories.

After the study, the researchers identified 120 illustrations that were rated the highest (20 for each category). After selecting the illustrations that most accurately represented each category, the researchers prepared an electronic questionnaire to conduct the study. In order to maintain randomness in the order of individual illustrations, a pseudo-random number generator was used when creating the survey.

The final version of the Morana Scale included 120 AI-generated illustrations selected through expert consensus. Participants were instructed to choose freely among the presented images. While formal data on completion time and dropout rates were not collected, the online format appeared feasible and well-tolerated. Scoring was based on cumulative selection patterns across predefined thematic clusters. No diagnostic cut-offs or normative interpretations were applied, as the instrument remains in an exploratory phase of development.

### 2.3. Study Population

The study included 204 adults aged 18–66 years (M = 30.34). The characteristics of the study group are presented in Table 1.

### 2.4. Ethical Considerations

The study was approved by the Bioethics Committee of the Medical University of Warsaw (no. AKBE/322/2024). Informed consent was obtained via checkbox prior to participation, following a clear explanation of the study’s purpose, anonymity, and voluntary nature. Participants were explicitly informed that the study does not offer diagnosis or psychological support, and were advised to discontinue if they felt distressed.

Given the sensitive nature of the constructs explored (e.g., psychological pain, suicidal ideation), the survey concluded with a standardized debriefing page. This page provided psychoeducational context and listed mental health resources, including national crisis hotlines and referral contacts. Participants were reminded that the survey was not intended for individuals in acute crisis and were encouraged to seek immediate external help if needed. As the study was anonymous and non-interventional, no real-time monitoring or follow-up procedures were possible.

### 2.5. Data Analysis

Statistical analyses were carried out using the Statistica 13.3 program enriched with the Plus Set licensed by the Medical University of Warsaw. In the course of the statistical analyses, a number of methods were used to accurately assess the effectiveness and reliability of the developed multidimensional scale for assessing the risk of destructiveness. First, factor analysis with Varimax rotation was carried out, which allowed to identify hidden structures in the data, reduce their dimensionality and simplify the interpretation of the results.

Then, in order to assess the reliability of the scale, Cronbach’s alpha tests were used, which made it possible to determine the internal consistency of individual subscales. In addition, Spearman’s rho correlations were used to analyze the relationships between variables, which allowed them to study nonlinear relationships in the data.

To assess the differences between the groups, the Mann–Whitney U test was used, which is appropriate for nonparametric comparisons. Finally, logistic regression was performed to analyse the effect of independent variables on the dependent variable, both in univariate and multivariate versions, which made it possible to investigate the interplay between different factors and the risk of destructiveness.

Subscale dichotomization (mean + 0.5 SD) was applied to facilitate exploratory classification in logistic models. We acknowledge that this approach reduces statistical power and introduces class imbalance. These thresholds are not intended to define clinical categories and should be interpreted cautiously.

In addition to analyses performed in Statistica 13.3 (Plus Set, licensed by the Medical University of Warsaw), selected psychometric and predictive procedures were implemented using Python (v3.11) with dedicated scientific libraries (e.g., NumPy, pandas, scikit-learn). This environment enabled precise computation of tetrachoric correlations, Parallel Analysis, McDonald’s omega, and ROC curve diagnostics, which are not natively supported in standard statistical packages. Python was also used to automate reliability estimation, simulate random eigenvalue distributions, and visualize logistic regression outcomes.

All predictive models were exploratory and aimed to identify internal associations within the instrument. No external validation was performed in the current sample.

All relationships, correlations and regressions and differences were statistically significant at *p* ≤ 0.05.

## 3. Results

### 3.1. Factor Extraction

In order to extract the subscales, a Varimax factor analysis was performed using the principal axis method. A minimum level of charging factors above the threshold of 0.3 was assumed. In the case of the first factor (depression and psychological pain subscale), which was charged by a larger number of items, the threshold was assumed at the level of 0.4. After a factor analysis, the initial assumption of the existence of 12 subscales was modified in the light of the data obtained. As a result, seven factors were initially identified, and the previous assumptions were revised. However, when analyzing the data obtained, it was noticed that factor 4, which referred to death and destruction, had individual items that did not match the others. In order to understand the observed inconsistencies, reference was made to the theoretical foundations of Freud’s concept of the death drive (Thanatos). As a result, it was found that the Destruction factor exhibits two properties: the desire for destruction and its sublimation. Therefore, it was decided that two separate subscales would be created to reflect alternative ways of processing the death drive. Items included in these factors were assigned on the basis of factor analysis, but also after a qualitative assessment made by the researchers, based on theoretical assumptions.

The qualitative analysis of the illustrations classified into individual categories, created within the framework of the eight factors, allowed us to give them working names, partly referring to the original theoretical assumptions. The resulting subscales have been predefined as: depression and psychological pain subscale, interest and curiosity subscale, aggression and impulsiveness subscale, destruction drive subscale, sublimation of destruction subscale, pleasure and fun subscale, closeness and love subscale, anxiety and sense of threat subscale.

Internal consistency was assessed using three complementary indices: Cronbach’s alpha based on a Pearson correlation matrix, Cronbach’s alpha based on a tetrachoric correlation matrix (iterative approximation), and McDonald’s omega total. The tetrachoric alpha provides a more accurate estimate of reliability for binary items, while omega accounts for the latent factor structure. Table 2 presents the reliability coefficients and item counts for each subscale.

Factorability of the data was assessed using the Kaiser-Meyer-Olkin (KMO) measure and Bartlett’s test of sphericity. The overall KMO value was 0.56, indicating minimally acceptable sampling adequacy for factor analysis. Item-level KMO values ranged from 0.28 to 0.77, suggesting variable shared variance across items. Bartlett’s test was statistically significant (χ^2^ = 14 617.93, *p* < 0.001), confirming the suitability of the correlation matrix for factor extraction. Due to near-singularity of the covariance matrix, the Moore–Penrose generalized inverse was applied, which is acceptable for large dichotomous datasets.

Parallel Analysis was conducted separately for each subscale (7–18 dichotomous items), revealing a predominantly unidimensional structure across the instrument. In all cases except one (“Sublimation of Destruction”), only the first eigenvalue exceeded its random counterpart, supporting the presence of a dominant latent factor. The “Sublimation of Destruction” subscale yielded a three-factor solution, suggesting multidimensionality and warranting further structural exploration. Item–total correlations were moderate to high (range: 0.32–0.73), and communalities from one-factor EFA were modest (mean range: 0.01–0.03), consistent with binary scaling. These results support the psychometric coherence of the subscales and justify their treatment as latent constructs, while highlighting the need for further dimensional analysis in selected domains.

Cross-loadings were reviewed and did not compromise interpretability. Items with low loadings or poor thematic coherence were removed during iterative refinement. The post hoc split of Destructive Drive factor was supported by a multimodal loading pattern and low inter-item correlations between thematic clusters, consistent with its three-factor structure in parallel analysis.

### 3.2. Exploratory Analyses

In the first stage, correlation analyses were carried out, which revealed the relationships between individual scales (see Table 3).

#### 3.2.1. Destructive Drive

The subscale of destructive drive positively correlates with the subscale of depression and psychological pain (rho = 0.48), the subscale of aggression and impulsiveness (rho = 0.44), the subscale of anxiety and sense of threat (rho = 0.25) and negatively with age (rho = −0.20). Anxiety is additionally correlated with depression and psychological pain (rho = 0.31) and aggression and impulsiveness (rho = 0.57). Difficulties in regulating the emotions associated with depression can lead to violent, uncontrollable behavior, and psychological discomfort can exacerbate destructive tendencies. In addition, people experiencing depression often experience an increased sense of threat and fear, which creates a state of increased mental tension and promotes impulsivity. As a result, the above-mentioned mental states can be considered potentially conducive to the pursuit of destruction. The risk of destructiveness appears to decrease with age, which puts younger people at higher risk.

#### 3.2.2. Sublimation of Destruction

The subscale of destruction sublimation positively correlates with the interest and curiosity subscale (rho = 0.76), the pleasure and fun subscale (rho = 0.55), the closeness and love subscale (rho = 0.60) and the aggression and impulsivity subscale (3) (rho = 0.28) and the anxiety and threat subscale (rho = 0.17). Sublimation of destruction is a mechanism that transforms negative impulses into more constructive actions. There is a clear link between sublimation and curiosity, playfulness, and closeness, which may suggest positive coping strategies. In contrast, weaker correlations with impulsivity and anxiety may indicate some elements of anxiety or emotional reactivity, but not their dominant influence.

### 3.3. Modelling Destructive Tendencies: Statistical Pathways to Risk and Transformation

Logistic regression analyses were conducted to better understand the dynamics of the psychological processes that promote destruction, as well as the mechanism by which destructive impulses are transformed into constructive actions. The division of the results into groups of high and low destructiveness and sublimation of destruction was made on the basis of the limit determined as the mean plus half of the standard deviation. Due to the unequal size of groups with low and high destructiveness and sublimation (tendency to destruction: 48:156; sublimation of destruction: 49:155), statistical weighting was used to increase sample representativeness, adjust the impact of individual observations and improve the comparability of results.

In order to better understand the dynamics of the processes involved in destruction and its sublimation, two types of logistic regression analyses were performed. First, univariate models were applied for each of the dependent variables (destruction drive and sublimation of destruction), allowing the assessment of the individual impact of individual predictors. Then, multivariate models were created, taking into account the broader context of the interplay of variables. Comparison of the results of both approaches allowed for a more precise interpretation of the mechanisms affecting the risk of destruction and its sublimation strategies. Table 4 presents a comparison of the results obtained for the dependent variables: destructive drive and sublimation of destruction, respectively.

#### 3.3.1. Comparison of Univariate and Multivariate Models

##### Destructive Drive

Depression and psychological pain are a strong predictor of destructive tendencies, both as a single variable and after adjusting for other factors. In both models, a large statistically significant effect is observed, although in the multivariate model the OR decreases slightly.

Interest and curiosity are a trait that can reduce the drive to destruction, but only after taking into account other variables. Although in the univariate model the effect is statistically insignificant, in the multivariate model it becomes moderate and has a protective character against destructive tendencies.

Aggression and impulsivity is also a statistically significant variable, although its impact decreases after taking into account other factors. In the univariate model, a large effect is observed, while in the multivariate model it weakens significantly.

There is no significant effect indicating a relationship between the sublimation of destruction and the pursuit of destruction.

Pleasure and fun also reveal some connection with destructive tendencies, although it is small. In the univariate model, the effect was very small, and in the multivariate model it increases slightly, but it still remains small and statistically insignificant.

Closeness and love reveals a protective effect against destruction, which is slightly stronger in the multivariate model, but still remains small and statistically insignificant.

Anxiety and a sense of threat lose their significance in the multivariate model, although in the univariate the effect is large.

In summary, the drive for destruction is motivated by depression, aggression, and anxiety, and weakened by interest and curiosity, although only in a multivariate model. The strongest predictor of destruction is depression and psychological pain. An increase in depression and psychological pain by one point increases the risk of seeking destruction by 38 in the univariate model and 33% in the multivariate model, respectively.

##### Sublimation of Destruction

Depression and psychological pain are not a significant variable in the context of sublimation of destruction. In the univariate model, the effect is small, and in the multivariate model, it disappears completely.

Interest and curiosity strongly influence the sublimation of destruction, both in the univariate and multivariate models, although in the latter the effect weakens slightly.

Aggression and impulsivity is a variable that creates a stable effect that is moderate, both in the univariate and multivariate models.

The pursuit of destruction does not have a significant impact on the sublimation of destruction. In a univariate model, the effect is weak but positive, while in a multivariate model, it becomes negative. This may suggest that the sublimation of destruction does not result from destructive tendencies, but may be an alternative form of dealing with them.

Pleasure and fun is an important factor in the sublimation of destruction. In the univariate model, the effect is large, although in the multivariate model it weakens slightly, and in both cases it remains statistically significant.

Closeness and love have a positive effect on the sublimation of destruction, although the effect that is large in the univariate model becomes moderate in the multivariate model.

Anxiety and a sense of threat is a less important variable. In a univariate model, it has a medium impact, and in a multivariate model, it decreases to a small one.

In summary, the sublimation of destruction is associated with interest and curiosity, closeness and love, and pleasure and fun. The most stable predictor of sublimation of destruction is interest and curiosity. An increase in this subscale by one point increases the chances of sublimation of destruction by 215% in the univariate model and 107% in the multivariate model.

**Figure 1:**
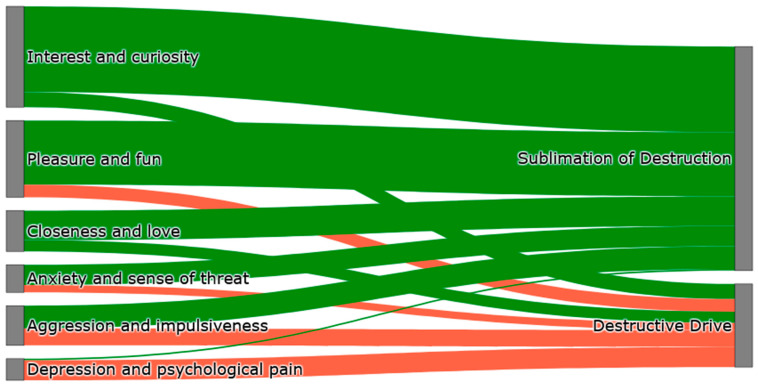
Sankey flow diagram illustrating internal associations between independent variables and destructive drive/sublimation in an exploratory multivariate model.

Multivariate model illustrating internal predictors of destructive drive and sublimation of destruction (exploratory classification only) is illustrated in Figure 1.

##### Age and Offspring

Additional regression analyses concerning the effect of age on destructive tendencies indicate that it is a protective factor against the tendency to destructive, although the effect is small and variable is not a key predictor of destructive tendencies. In the multivariate model, after taking into account the number of children, the effect is enhanced. The odds ratio suggests that with each year of life, the risk of seeking destruction decreases by 4.1% in the univariate model and by 5.3% in the multivariate model. The effect of age on sublimation of destruction is minimal and statistically insignificant.

Having children is not a statistically significant variable in the context of sublimation of destruction in any model. The results of logistic regression for the age and number of children in the context of destructive drive and its sublimation are presented in Table 5.

#### 3.3.2. ROC Curve Analysis

In order to explore the internal discrimination capacity of individual subscales and to identify tentative thresholds for classifying relative risk levels of destruction and its sublimation, ROC curve analyses were performed. The results are presented in Table 6.

Analysis of the ROC curve for depression and psychological pain suggests that this subscale has relatively strong internal discrimination capacity for differentiating higher and lower levels of destructive drive, the cut-off point is 3, indicating that people with higher depression scores have a higher risk of destruction. The parameters of the model indicate a relatively balanced ability to correctly identify people who seek destruction and those who do not.

The ROC curve for aggression and impulsivity indicates moderate internal discrimination between individuals with higher and lower scores on destructive drive. The properties of the model suggest that it is slightly better at correctly excluding people without seeking destruction. The cutoff point for the destruction classification is 1. The model is able to identify people prone to destruction and has a relatively low level of false positives, indicating high classification accuracy.

Anxiety and a sense of threat show limited internal discrimination in relation to destructive drive, weaker than in the case of the previous two factors. The cutoff point for predicting destruction is 2. The model is much better at ruling out the drive for destruction than at correctly identifying it, and some level of false classifications can be expected.

The ROC curve for the interest and curiosity subscale yielded a high AUC value, indicating strong internal discrimination. The model parameters suggest balanced sensitivity and specificity within the current sample of the model. The cut-off point is 2.

The pleasure and play subscale demonstrated good internal discrimination. The model shows balanced sensitivity and specificity, and the cut-off point for identifying individuals sublimating destruction is 1.

Analysis of the ROC curve for closeness and love suggests strong internal discrimination in relation to sublimation of destruction. The model is characterized by good balance and effectiveness. The factor may allow for effective differentiation of people who are more likely to sublimate destructive impulses. The cut-off point of 1 reflects an exploratory threshold and does not imply diagnostic classification.

AUC values obtained from ROC analyses reflect internal discrimination between relatively high and low scorers on the same instrument. As no external criterion was used, these results do not establish diagnostic validity.

#### 3.3.3. Intergroup Differences

The last stage of the analysis included the assessment of intergroup differences in the participation of the respondents in psychotherapy, the use of psychiatric treatment, having a psychiatric diagnosis and professional activity.

The subscale of destructive drive was the only one that differentiated the respondents in terms of participation in psychotherapy (U = 3997.50, *p* < 0.03), psychiatric treatment (U = 4151.50, *p* < 0.03), psychiatric diagnosis (U = 4124.50, *p* < 0.02) and professional activity. Respondents who participated in psychotherapy, psychiatric help and had a psychiatric diagnosis obtained higher results than those who did not use such forms of help. On the other hand, in the context of professional activity, learners showed a higher level of striving for destruction than economically active people (U = 3598.00, *p* < 0.01). However, no significant differences were found in the case of students and employed people and the unemployed.

## 4. Discussion

The study presented the process of construction and initial validation of the psychometric Morana Scale, a projective method for assessing the risk of destructiveness, developed using artificial intelligence. In accordance with the original theoretical assumptions, the initial subscales were based on clinical determinants of suicide, such as depression, aggressiveness, impulsivity, anxiety, psychological pain and fantasies about death. The authors’ primary goal was to create a tool for assessing the risk of suicide, but the results in the course of the analyses prompt a reconsideration of this idea and its potential for psychological differentiation rather than diagnostic classification. The final shape of the subscales indicates that the method is rather intended to differentiate people prone to destruction from those who subject aggressive impulses to sublimation, and thus reduce the risk of threatening activities in favour of developmental activities consistent with social norms.

In the course of our analyses, we observed that several identified factors appear to conceptually align with the emotional systems described by Jaak Panksepp, who distinguished seven primary affective networks located in subcortical brain structures [57,58,59,60,61]. While this theoretical correspondence is intriguing, we emphasize that the current study does not include neurobiological or behavioral data to empirically support such mapping. Therefore, we present these associations as hypothetical and theory-driven, pending future validation.

Specifically, six of the eight factors show thematic resemblance to Panksepp’s systems: depression and psychological pain to PANIC/GRIEF; interest and curiosity to SEEKING; aggression and impulsivity to RAGE; pleasure and play to PLAY; closeness and love to CARE; and fear and threat to FEAR. The LUST system was not directly reflected. Two remaining factors—destructive drive and its sublimation—did not align with Panksepp’s framework and may reflect distinct psychological constructs related to maladaptive or transformative processes. These interpretations remain speculative and require further empirical investigation.

To avoid overinterpretation, we acknowledge that the current findings reflect internal psychometric relationships and do not yet establish external diagnostic or prognostic validity. While the observed associations align with psychoanalytic and neurobiological models, they remain speculative and require empirical testing. In this regard, our method shares conceptual features with performance-based approaches, where validity depends on rigorous, multi-criterion evidence rather than theoretical correspondence. As Mihura et al. emphasize, such instruments require convergent, discriminant, criterion-related, and incremental validation to support clinical utility [62].

Accordingly, future research will pursue a structured program of external validation, including comparisons with established clinical instruments (e.g., SBQ-R, C-SSRS, BDI-II), behavioral outcomes (e.g., hospitalization, self-harm history), and prospective follow-up data. This will enable us to assess the predictive and incremental validity of the Morana Scale and refine its theoretical positioning.

The presented study revealed a number of connections between the pursuit of destruction and its sublimation, and individual variables representing specific mental states. The observed associations, visible between the subscales of the Morana Scale, are consistent with current scientific knowledge, which suggests potential for future predictive applications in suicide risk assessment, pending external validation. In our analyses, the drive for destruction is most influenced by depression and mental pain, but also by aggression and impulsivity, as well as anxiety and a sense of threat. The literature commonly associates depression with a high risk of suicidal thoughts and behaviours [63], as well as impulsivity and aggressiveness [64,65] and anxiety disorders [66].

A clinical example illustrating the relationship between these traits and destructiveness is borderline personality disorder. The above range of mental states in this disorder is conducive to an increased risk of suicide. People with borderline disorder often experience intense and unstable feelings, have difficulty regulating emotions, and take impulsive, aggressive actions, including repeated acts of self-harm and suicidal behavior [67].

On the other hand, the sublimation of destruction is a psychological defense mechanism that allows negative impulses to be transformed into constructive actions. The analysis showed a relationship between the sublimation of destruction and openness to new experiences and curiosity, the search for pleasure and fun, as well as closeness and love. Research on the relationship between openness to new experiences and health provides diverse conclusions. This trait can contribute to beneficial effects, but also to negative effects. Bresin and Hunt showed that there is a positive relationship between openness to new experiences and non-suicidal self-harm [68]. At the same time, this trait is a protective factor in relation to all-cause mortality [69]. Some researchers assume that a low level of openness to new experiences may promote suicidal behavior due to reduced adaptive abilities due to cognitive rigidity, a limited repertoire of behaviors, and a strongly defined identity [70]. On the other hand, people with a high level of openness to new experiences are characterized by a greater ability to think divergently, innovative problem-solving strategies, and creative achievement [71]. This may explain the observation made in our study that interest and curiosity are the strongest predictors of sublimation of destruction.

Another factor that influences the constructive transformation of destructive impulses is pleasure and play. As Davidson et al. writes, such positive experiences can play an important role in the recovery of people with mental disorders [72]. The experience of pleasure is a complex neurobiological phenomenon based on reward pathways and limbic activity. It can support cognitive processes, productivity, and health, but at the same time lead to negative behaviors. Moderate pleasurable experiences can increase biological flexibility and health, but seeking out artificial stimulants can be detrimental and lead to addiction [73]. This duality was also revealed in the analyses of the results presented in our study, where the subscale of pleasure and play was associated not only with the sublimation of destruction, but also with the pursuit of it, as opposed to the protective influence of interest and curiosity as well as closeness and love.

Closeness and love are the last of the three predictors of sublimation of destruction identified in the process of constructing the Morana Scale. Research shows that the health-promoting function of closeness and love, which reduces the risk of destruction, is a decrease in cortisol levels, activation of the reward system, reduction in the risk of depressive and anxiety disorders, increase mental resilience and improve the physical condition of the body [74]. The importance of this type of experience is crucial from the first moments of life, when the stress axis (HPA) and the oxytocin system are intensively activated. If the first physical contact between mother and baby occurs immediately after birth, the oxytocin system, which has anti-stress potential, becomes more pronounced, which in turn has a long-term impact on the baby’s health [75]. From the perspective of psychological processes, later in life, love and closeness can create a safe environment in which the emotional exchange of difficult affects can proceed in a more stable way based on sublimation rather than violent destructive reactions [76].

The mechanism of sublimation of destruction may be a key protective factor in the context of suicidal risk and other forms of self-harm. The observed associations with health-promoting competences can be related to the Freudian definition of health, described as the ability to work and love [77]. The mechanism of sublimation of destruction would be a key aspect in this case in terms of transforming potentially harmful impulses from biological, primary layers of experience into more mature ones conducive to building interpersonal relationships and professional productivity.

With regard to Melanie Klein’s concept, it can be assumed that the ability to transform hateful and destructive impulses into constructive forms is related to the quality of the relationship with the original object, usually the mother. It is how the child experiences the mother, both in good and bad aspects, that shapes the ability to cope with his own aggression. According to Klein’s assumptions, sublimation is a mental process that allows aggression and hatred to be transformed into creative and prosocial activities, which in turn determines mental health [78,79,80].

Lacan, on the other hand, understood sublimation as the transfer of the drive from its original goal to the space of more symbolic satisfaction. According to his thought, the primordial energy can be transformed into forms for creation and development, e.g., in the form of art, literature or philosophy. Following Lacan’s thought, where sublimation finds reference to language and symbols, destruction can be transformed into art and literature that explore the dark aspects of the human mind [81,82].

The last aspect of the analysis included a comparison of the respondents in terms of all subscales in the context of professional activity, the use of psychological and psychiatric help, as well as having a psychiatric diagnosis. The differences were revealed only in the context of striving for destruction. Learners showed stronger inclinations towards destruction than working people. This may be related to age. A large study spanning 90 countries found that young learners show a higher risk of suicide than any other age group [83]. Similarly, a study by Yan et al. [84] indicates that school-aged and college-aged people may be at higher risk of suicidal thoughts than older working people. Although only adults participated in our study, they were still relatively the youngest of the entire study group.

Interestingly, people who received the help of a psychologist or psychiatrist, and those who had a diagnosis, showed a higher level of destructive drive than those who were not treated. This may be due to the fact that people with stronger destructive impulses are more likely to come into contact with a mental health professional at some point in their lives, e.g., as a result of crisis intervention, hospitalization or other activities related to their risky or threatening behaviour. Research confirms that not all people in mental crisis seek professional help, often delay the decision to seek treatment, and come to psychiatric care not on their own initiative, but rather as a result of contact with health care structures [85].

The presented study made it possible to construct a tool, isolate factors and assess their reliability. From the perspective of theoretical foundations, the method draws inspiration from classical psychoanalytic assumptions regarding the death drive and engages with contemporary theoretical models of emotional and motivational processes, including neurobiological perspectives. However, these connections remain conceptual and require empirical validation. The obtained results prove satisfactory psychometric parameters of the method and the predictive value of the identified subscales in relation to the pursuit of destruction and its sublimation.

In the next stage, research is planned to assess the validity of the external scale in relation to indicators of general suicidality risk, specific risk factors consistent with theoretical assumptions, as well as Panksepp’s emotional systems associated with scales in the final analyses.

The study has some limitations. Initial validation was performed on a limited sample of subjects, with a significant predominance of female subjects, which may limit the possibility of generalizing the results to a wider clinical population. In addition, limitations in sample size and the use of convenience samples may have affected the stability of the results and the consistency of the factor structure [86]. A controversial aspect of the presented study may also be the projective nature of the method being developed, which is based on an interpretative mechanism that is susceptible to the influence of subjective factors on the part of both the researcher and the subject [87]. This type of diagnosis uses ambiguous symbolic stimuli, which makes standardization difficult and raises doubts among some researchers and clinicians [11,88]. This may be related to the difficulty of unambiguously linking symbolic visual content with specific suicide risk factors. In this respect, the projective formula, although allowing access to more unconscious content, is in itself considered insufficient [89].

The integration of AI-generated content into mental health assessment tools raises important questions about bias, explainability, and human agency. These aspects were not formally evaluated in the current study but will be addressed in future work through bias audits, participant feedback, and alignment with emerging regulatory standards [90,91].

Our findings reflect internal psychometric structure and associations. Future studies will incorporate external clinical measures (e.g., C-SSRS, BDI-II, SBQ-R, NSSI history) to assess criterion validity and improve generalizability. Confirmatory modeling (e.g., CFA/ESEM with WLSMV, IRT, DIF) will also be conducted in larger and independent samples.

The current version of the scale may be time-consuming in clinical or longitudinal contexts. Future iterations will explore short-form versions and computerized adaptive testing (CAT) contingent on item-level IRT parameter estimation and external validation.

Although the COSMIN Risk of Bias checklist for Patient-Reported Outcome Measures [92] was not formally applied, this limitation reflects the exploratory nature of the instrument. Future validation studies will incorporate COSMIN-based criteria to improve methodological transparency and comparability.

## 5. Conclusions

- The satisfactory psychometric properties of the tool suggest its potential to support clinical assessment, especially in identifying patterns associated with self-aggressive tendencies. However, its diagnostic utility requires further external validation.
- The constructs of destructive drive and its sublimation appear to reflect unconscious processes, but their diagnostic relevance remains to be empirically established.
- Interest and curiosity, pleasure and fun, and closeness and love support the mechanisms of sublimation of destruction, suggesting the need to develop therapeutic strategies based on positive motivation rather than just reducing risky behaviors.
- The results of the presented research are a point of contact between the psychoanalytic approach and modern neurobiological knowledge, which opens up the possibility of integrative therapeutic methods.
- The next step in the presented study is to assess the external validity of the Morana Scale in order to determine its usefulness in clinical practice, including the assessment of suicidal risk. It is also advisable to investigate the possibility of adapting the tool to different clinical groups in order to improve the effectiveness of interventions in different therapeutic contexts.

## Figures and Tables

**Table 1:** Characteristics of the study group.

Variable	Category	N	%
Sex	Woman	156	76.47
	Man	48	23.52
Age	18–66		
Marital status	Free	136	66.66
	Marriage	55	26.96
	Divorce	11	5.39
	Widowhood	2	0.98
Number of children	0	157	76.96
	1	16	7.84
	2	23	11.27
	3	7	3.43
	More	1	0.49
Education	Basic	1	0.49
	Grammar school	1	0.49
	Essential professional	3	1.47
	Average	76	37.25
	Higher	123	60.29
Domicile	Village	34	16.66
	A city below 100 thousand	33	16.17
	City 100–500 thousand	18	8.82
	City over 500 thousand	119	58.33
Professional activity	Education	80	39.21
	Professional career	116	56.86
	Unemployment	8	3.92
Psychiatric treatment currently or in the past	No	82	40.19
	Yes	122	59.80
Psychotherapy now or in the past	No	74	36.27
	Yes	130	63.72
Psychiatric diagnosis	No Yes	83 121	40.68 59.32
	Reported disorders:		
	Affective Disorder F30–F39 Psychotic disorders F20–F29 Personality Disorders F60–F69 Anxiety Disorders F40–F49	98 3 4 16	48.04 1.47 1.96 7.83
The impact of the diagnosis on current well-being	Not applicable	74	36.27
	No	30	14.70
	Yes	100	49.01

**Table 2:** Internal consistency and item count for subscales.

Subscale	Number of Items	Alfa Cronbach (Pearson)	Alfa Cronbach (Tetrachoric)	Omega McDonald (Total)
(1) Depression and Psychological Pain	18	0.85	0.857	0.947
(2) Interests and Curiosity	15	0.87	0.89	0.94
(3) Aggression and Impulsiveness	14	0.82	0.83	0.93
(4a) Destructive Drive	8	0.75	0.75	0.898
(4b) Sublimation of Destruction	11	0.73	0.73	0.92
(5) Pleasure and Fun	14	0.82	0.83	0.93
(6) Closeness and Love	7	0.71	0.72	0.88
(7) Anxiety and Sense of Threat	9	0.74	0.76	0.90

**Table 3:** Analysis of Spearman’s rho correlation between individual factors and the age and number of children.

Variable	P1	P2	P3	P4a	P4b	P5	P6	P7	Age	Number of Children
P1	1	−0.06	0.39	0.48	0.09	0.05	−0.03	0.31	−0.08	−0.02
P2	−0.06	1	0.12	−0.06	0.76	0.56	0.65	0.07	0.11	−0.01
P3	0.39	0.12	1	0.49	0.28	0.07	0.09	0.57	−0.05	−0.02
P4a	0.48	−0.06	0.44	1	0.05	0.09	−0.02	0.25	−0.2	−0.08
P4b	0.09	0.76	0.28	0.05	1	0.55	0.6	0.17	0.09	−0.01
P5	0.05	0.56	0.07	0.09	0.55	1	0.4	0.03	−0.02	−0.08
P6	−0.03	0.65	0.09	−0.02	0.6	0.4	1	0.05	0.03	0.1
P7	0.31	0.07	0.57	0.25	0.17	0.03	0.05	1	0.04	0.03
Age	−0.08	0.11	−0.05	−0.2	0.08	−0.02	0.03	0.04	1	0.6
Number of children	−0.02	−0.01	−0.02	−0.08	−0.01	−0.08	0.1	0.03	0.6	1

P1 = subscale of depression and psychological pain, P2 = subscale of exploring the world/interest in reality, P3 = subscale of aggression and impulsiveness, P4a = subscale of destructive drive, P4b = subscale of sublimation of destruction, P5 = subscale of pleasure and fun, P6 = subscale of closeness and love, P7 = subscale of anxiety and sense of threat.

**Table 4:** Comparison of univariate and multivariate models for the pursuit of destruction and sublimation of destruction.

**Destructive Drive**				
**Factor**	**Single-Variate Model (B, SE, Wald χ^2^, *p*, OR, 95% CI)**	**Effect Size**	**Multivariate Model (B, SE, Wald χ^2^, *p*, OR, 95% CI)**	**Effect Size**
Depression and psychological pain	B = 0.33, SE = 0.05, Wald χ^2^ = 47.57, *p* = 0.00, OR = 1.39, 95% CI [1.26–1.52]	Big impact	B = 0.29, SE = 0.05, Wald χ^2^ = 32.77, *p* = 0.00, OR = 1.33, 95% CI [1.21–1.47]	Big impact
Interest and curiosity	B = −0.05, SE = 0.05, Wald χ^2^ = 1.02, *p* = 0.31, OR = 0.95, 95% CI [0.87–1.05]	Lack	B = −0.29, SE = 0.14, Wald χ^2^ = 4.47, *p* = 0.03, OR = 0.75, 95% CI [0.57–0.98]	Medium (protective) effect
Aggression and impulsiveness	B = 0.52, SE = 0.11, Wald χ^2^ = 23.81, *p* = 0.00, OR = 1.68, 95% CI [1.36–2.06]	Big impact	B = 0.25, SE = 0.14, Wald χ^2^ = 3.36, *p* = 0.07, OR = 1.28, 95% CI [0.98–1.68]	Small effect
Sublimation of destruction	B = 0.10, SE = 0.07, Wald χ^2^ = 2.35, *p* = 0.13, OR = 1.11, 95% CI [0.97–1.26]	Lack	B = 0.13, SE = 0.13, Wald χ^2^ = 1.02, *p* = 0.31, OR = 1.14, 95% CI [0.88–1.48]	Lack
Pleasure and fun	B = 0.14, SE = 0.07, Wald χ^2^ = 3.67, *p* = 0.06, OR = 1.15, 95% CI [1.00–1.32]	Lack	B = 0.19, SE = 0.13, Wald χ^2^ = 2.14, *p* = 0.14, OR = 1.21, 95% CI [0.94–1.57]	Small effect
Closeness and love	B = −0.11, SE = 0.10, Wald χ^2^ = 1.32, *p* = 0.25, OR = 0.89, 95% CI [0.73–1.08]	Low (protective) effect	B = −0.21, SE = 0.14, Wald χ^2^ = 2.07, *p* = 0.15, OR = 0.81, 95% CI [0.61–1.08]	Low (protective) effect
Anxiety and sense of threat	B = 0.44, SE = 0.10, Wald χ^2^ = 18.07, *p* = 0.00, OR = 1.55, 95% CI [1.27–1.91]	Big impact	B = 0.11, SE = 0.14, Wald χ^2^ = 0.65, *p* = 0.42, OR = 1.12, 95% CI [0.85–1.48]	No effect
**Sublimation of Destruction**				
**Factor**	**Single-Variate Model (B, SE, Wald χ^2^, *p*, OR, 95% CI)**	**Effect Size**	**Multivariate Model (B, SE, Wald χ^2^, *p*, OR, 95% CI)**	**Effect Size**
Depression and psychological pain	B = 0.07, SE = 0.03, Wald χ^2^ = 4.74, *p* = 0.03, OR = 1.07, 95% CI [1.01–1.14]	Lack	B = 0.03, SE = 0.07, Wald χ^2^ = 0.14, *p* = 0.71, OR = 1.03, 95% CI [0.90–1.17]	Lack
Interest and curiosity	B = 1.15, SE = 0.13, Wald χ^2^ = 75.97, *p* = 0.00, OR = 3.15, 95% CI [2.43–4.09]	Big impact	B = 0.89, SE = 0.15, Wald χ^2^ = 33.24, *p* = 0.00, OR = 2.43, 95% CI [1.80–3.29]	Big impact
Aggression and impulsiveness	B = 0.30, SE = 0.08, Wald χ^2^ = 13.87, *p* = 0.00, OR = 1.35, 95% CI [1.15–1.58]	Medium effect	B = 0.32, SE = 0.16, Wald χ^2^ = 4.20, *p* = 0.04, OR = 1.38, 95% CI [1.01–1.88]	Medium effect
Destructive drive	B = 0.12, SE = 0.07, Wald χ^2^ = 3.06, *p* = 0.08, OR = 1.13, 95% CI [0.99–1.29]	Lack	B = −0.21, SE = 0.16, Wald χ^2^ = 1.71, *p* = 0.19, OR = 0.81, 95% CI [0.59–1.11]	Small effect
Pleasure and fun	B = 1.13, SE = 0.16, Wald χ^2^ = 51.54, *p* = 0.00, OR = 3.08, 95% CI [2.26–4.20]	Big impact	B = 0.73, SE = 0.20, Wald χ^2^ = 13.41, *p* = 0.00, OR = 2.07, 95% CI [1.40–3.06]	Big impact
Closeness and love	B = 1.23, SE = 0.17, Wald χ^2^ = 55.32, *p* = 0.00, OR = 3.43, 95% CI [2.48–4.76]	Big impact	B = 0.40, SE = 0.19, Wald χ^2^ = 4.41, *p* = 0.04, OR = 1.49, 95% CI [1.03–2.18]	Medium effect
Anxiety and sense of threat	B = 0.36, SE = 0.09, Wald χ^2^ = 14.66, *p* = 0.00, OR = 1.43, 95% CI [1.19–1.73]	Medium effect	B = 0.29, SE = 0.20, Wald χ^2^ = 2.08, *p* = 0.15, OR = 1.34, 95% CI [0.90–2.00]	Small effect

**Table 5:** Analysis of univariate and multivariate logistic regression for dependent variables: destructive drive and sublimation of destruction in relation to independent variables: age and number of children.

**Destructive Drive**				
**Variable**	**Single-Variate Model (B, SE, Wald χ^2^, *p*, OR, 95% CI)**	**Effect Size**	**Multivariate Model (B, SE, Wald χ^2^, *p*, OR, 95% CI)**	**Effect Size**
Age	B = −0.04, SE = 0.01, Wald χ^2^ = 11.86, *p* = 0.00, OR = 0.96, 95% CI [0.94–0.98]	Poor	B = −0.05, SE = 0.02, Wald χ^2^ = 13.68, *p* = 0.00, OR = 0.95, 95% CI [0.92–0.98]	Poor
Number of children	B = 0.01, SE = 0.10, Wald χ^2^ = 0.01, *p* = 0.94, OR = 1.01, 95% CI [0.83–1.23]	Lack	B = 0.24, SE = 0.15, Wald χ^2^ = 2.58, *p* = 0.11, OR = 1.28, 95% CI [0.95–1.72]	Lack
**Sublimation of Destruction**				
**Variable**	**Single-Variate Model (B, SE, Wald χ^2^, *p*, OR, 95% CI)**	**Effect Size**	**Multivariate Model (B, SE, Wald χ^2^, *p*, OR, 95% CI)**	**Effect Size**
Age	B = 0.01, SE = 0.01, Wald χ^2^ = 1.10, *p* = 0.29, OR = 1.01, 95% CI [0.99–1.03]	Lack	B = 0.01, SE = 0.01, Wald χ^2^ = 0.38, *p* = 0.54, OR = 1.01, 95% CI [0.98–1.03]	Lack
Number of children	B = 0.12, SE = 0.10, Wald χ^2^ = 1.32, *p* = 0.25, OR = 1.13, 95% CI [0.92–1.38]	Lack	B = 0.09, SE = 0.11, Wald χ^2^ = 0.64, *p* = 0.42, OR = 1.09, 95% CI [0.88–1.36]	Lack

**Table 6:** Summary of ROC analysis results for individual subscales.

**Destructive Drive**										
**Variables**	**AUC**	**SE**	**Cl 95%**	**Cut-off Point**	**Sensitivity**	**Specificity**	**Accuracy**	**Positive Predictive Value**	**Negative Predictive Value**	**Youden Index**
Depression and psychological pain	0.78 *	0.04	[0.71; 0.86]	3	0.75	0.65	0.67	0.40	0.89	0.40
Aggression and impulsiveness	0.72 *	0.04	[0.63; 0.80]	1	0.69	0.72	0.72	0.43	0.88	0.41
Anxiety and sense of threat	0.62 **	0.05	[0.52; 0.72]	2	0.40	0.90	0.78	0.56	0.83	0.30
**Sublimation of Destruction**										
**Variables**	**AUC**	**SE**	**Cl 95%**	**Cut-off Point**	**Sensitivity**	**Specificity**	**Accuracy**	**Positive Predictive Value**	**Negative Predictive Value**	**Youden Index**
Interest and curiosity	0.90 *	0.03	[0.85; 0.95]	2	0.84	0.87	0.86	0.67	0.94	0.71
Pleasure and fun	0.79 *	0.04	[0.71; 0.87]	1	0.76	0.76	0.76	0.50	0.91	0.52
Closeness and love	0.84 *	0.04	[0.77; 0.91]	1	0.84	0.80	0.81	0.57	0.94	0.64

* *p* < 0.00001; ** *p* < 0.02.

## Data Availability

The data supporting the findings of this study, including the full set of Morana Scale illustrations, are openly available in Zenodo at https://zenodo.org/records/15652742 (accessed on 12 June 2025). A preprint of the study is also available at Preprints (DOI: https://doi.org/10.20944/preprints202505.1276.v1 (accessed on 15 May 2025)), providing early access to the methodology and validation process.
